# Field Effect Transistor Based on Layered NiPS_**3**_

**DOI:** 10.1038/s41598-018-26522-1

**Published:** 2018-06-05

**Authors:** Ramesh Naidu Jenjeti, Rajat Kumar, Muthu P. Austeria, S. Sampath

**Affiliations:** Department of Inorganic and Physical Chemistry, Indian Institute of Science, Bangalore, 560012 India

## Abstract

Layered metal phosphochalcogenides of molecular formula, MPX_3_ (M = Mn, Fe, Co, Ni, etc and X = S, Se) have been emerging as new class of semiconductors towards various catalytic and optoelectronic applications. The low cleavage energy associated with these layered chalcogenides may lead to devices with very thin semiconductor channels. Herein, we report the first successful fabrication of field effect transistor (FET) using layered NiPS_3_ that reveals n-type semiconducting behavior. Devices using bulk and few-layer NiPS_3_ with gold contacts show on/off ratios of ~10^3^–10^5^ at 25 °C. The device characteristics reveal an increase in on-state current with decrease in threshold voltage and the Schottky barrier height is extracted to be 112 meV. Density functional theory calculations reveal various parameters that affect electron/hole doping in the layered phosphochalcogenide material.

## Introduction

Among the 2-dimensional layered materials, graphene has received considerable attention due to its ultrahigh mobility and tunability of layer thickness^1,2^. However, the zero band gap of pristine graphene limits its use in optoelectronics and other applications. Transition metal dichalcogenides (TMDs) such as MoS_2_ form the next class of well-studied compounds with certain band gap tunability^3–12^. Bulk MoS_2_ is semiconducting in nature with an indirect band gap of ~1.2–1.4 eV^13^, while mono-layer MoS_2_ possesses direct band gap of 1.8 eV^14^. This has led to various fundamental studies in the areas of electronic, optoelectronic and ultrasensitive sensors with atomically thin MoS_2_ membranes^15–23^. Though the TMDs (MoS_2_, WSe_2_) have shown high on/off ratios with tunable band gap in the visible wavelength range^24,25^, the low carrier mobility is of concern. Recently, phosphorene has been looked at, as a potential candidate for optoelectronic, electronic devices and sensors^26–28^, owing to its high on/off ratio coupled with high carrier mobility^29,30^. The disadvantage though is its stability that is still being tackled. Both TMDs and black phosphorus possess small band gaps thus restricting their applications in optoelectronics using light of short wavelength field effect transistors (FETs)^20,26^.

Continuous search for new 2D-materials has recently led to a well-studied class of bulk layered semiconducting metal phosphotrichalcogenides with formula MPX_3_ (M = Ni, Fe, Mn, Co, V, Zn etc; X = S and Se). This class of layered compounds have been wellexplored in the latter half of 20^th^ century towards understanding their crystal structure and intercalation properties^31–39^. However, little is known as for as few layer MPX_3_ materials are concerned and recently, this area has been attracting considerable attention^40–42^ particularly towards catalysis^43–47^ and UV photodetector^48^. A recent review^49^ highlights the importance of this class of materials and their multifunctionality. The MPX_3_ family of materials possesses wide variation of band gap values from 1.3 eV to 3.5 eV. The cleavage energy of MPX_3_ is reported to similar and in certain cases, lower than that of graphene and TMDs^41,50^. Depending on the nature of metal ion, MPX_3_ family of compounds may open up ways to fabricate field effect transistors which is still a missing link in the current literature. A recent report on the first principle calculations on MnPSe_3_ reveals transformation from anti-ferromagnetic semiconductor to ferromagnetic half-metal by carrier doping^37^. Raman spectroscopic studies on certain stable MPX_3_ compounds has been recently reported^40,41^. However, electronic devices based on these classes of materials such as field effect transistors have not been explored in the literature so far.

Among the MPX_3_ family of semiconductors, NiPS_3_ is a layered ternary metal thiophosphate with monoclinic crystal system containing two molecular formula units per unit cell (Ni_2_P_2_S_6_), with point group, C2/m. NiPS_3_ is comprised of layers of covalently bonded units of (P_2_S_6_)^4−^ bipyramids with honeycomb arrangement of divalent nickel ions. The sulfur atoms are hexagonally arranged along the c-axis in ABCABC sequence. The inter layers are connected through weak van der Waals forces similar to other layered 2D-materials such as TMDs. Hence, it is possible to separate the layers by mechanical exfoliation. Bulk NiPS_3_ is anti-ferromagnetic with magnetic moment of 3.9 BM^51^. The reported band gap of bulk NiPS_3_ is ~1.6 eV^43^.

In the present study, we have synthesized fairly large sized, oriented crystals of NiPS_3_ using a high temperature solid state method and explored its use in FETs. The material is thoroughly characterized using X-ray diffraction (XRD), Raman spectroscopy, transmission electron microscopy (TEM) and atomic force microscopy (AFM). Field effect transistors (FETs) have been fabricated and temperature dependent electrical transport measurements have been carried out. Density functional theory (DFT) calculations reveal the possible parameters that help understand the carrier-type observed in the electrical transport studies.

## Result and Discussion

**Figure 1:**
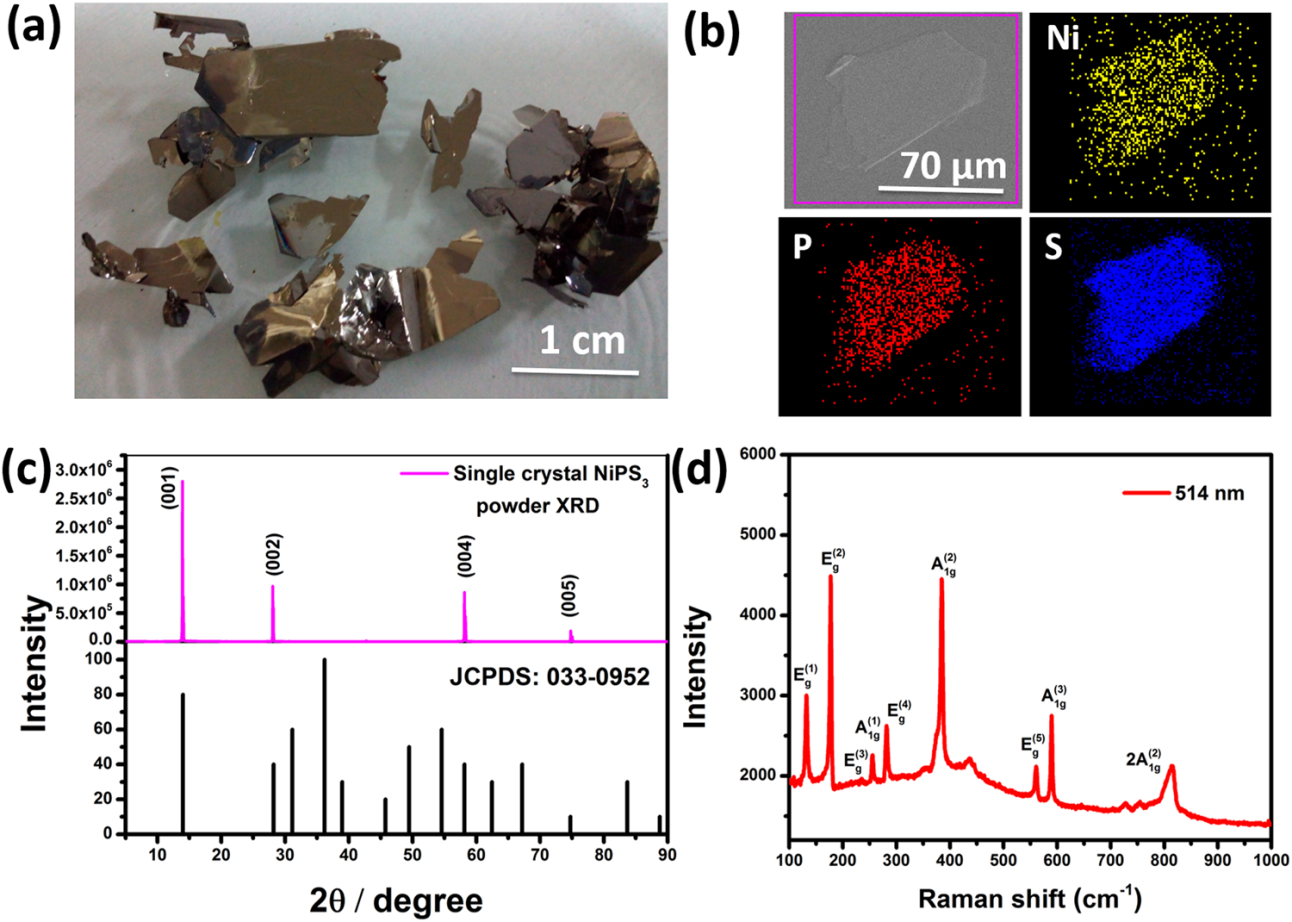
(**a)** Photographic images of NiPS_3_ crystals. (**b)** Scanning electron microscopic image with elemental mapping of mechanically exfoliated few layer NiPS_3_ on Si/SiO_2_ along with (**c)**. XRD pattern of the bulk crystals and the corresponding standard pattern. (**d)** Raman spectrum of NiPS_3_ crystals obtained with 514 nm excitation laser.

Highly oriented crystals of NiPS_3_ obtained in the present study are shiny black in colour with high crystallinity and the X-ray diffraction pattern shows very high orientation in the (00 l) direction. Figure 1(a–c) shows the optical images, scanning electron microscopic image with elemental mapping and the XRD pattern of large sized crystals. The Raman spectrum of the bulk material (Fig. 1d) shows a band at 253 cm^−1^ which is assigned to the A_1g_^(1)^ mode. The high intense peak at 176 cm^−1^ is due E_g_^(2)^ vibration and the one observed at 384 cm^−1^ is due to symmetric stretching vibration of P-S bond in the P_2_S_6_ units (assigned as A_1g_^(2)^). The bands at 236 cm^−1^, 280 cm^−1^, 560 cm^−1^ and 588 cm^−1^ are assigned to E_g_^(3)^, E_g_^(4)^, E_g_^(5)^ and A_1g_^(3)^ modes^40,51,52^. The bulk electrical conductivity has been measured to be 1.64 × 10^−7^ S/cm at 25 °C. TMDs such as 2H MoS_2_, MoSe_2_, WSe_2_ and MoTe_2_ show values of 3 × 10^−2^, 2 × 10^−1^, 6 and 1.8 S/cm respectively at 25 °C^53^.

**Figure 2:**
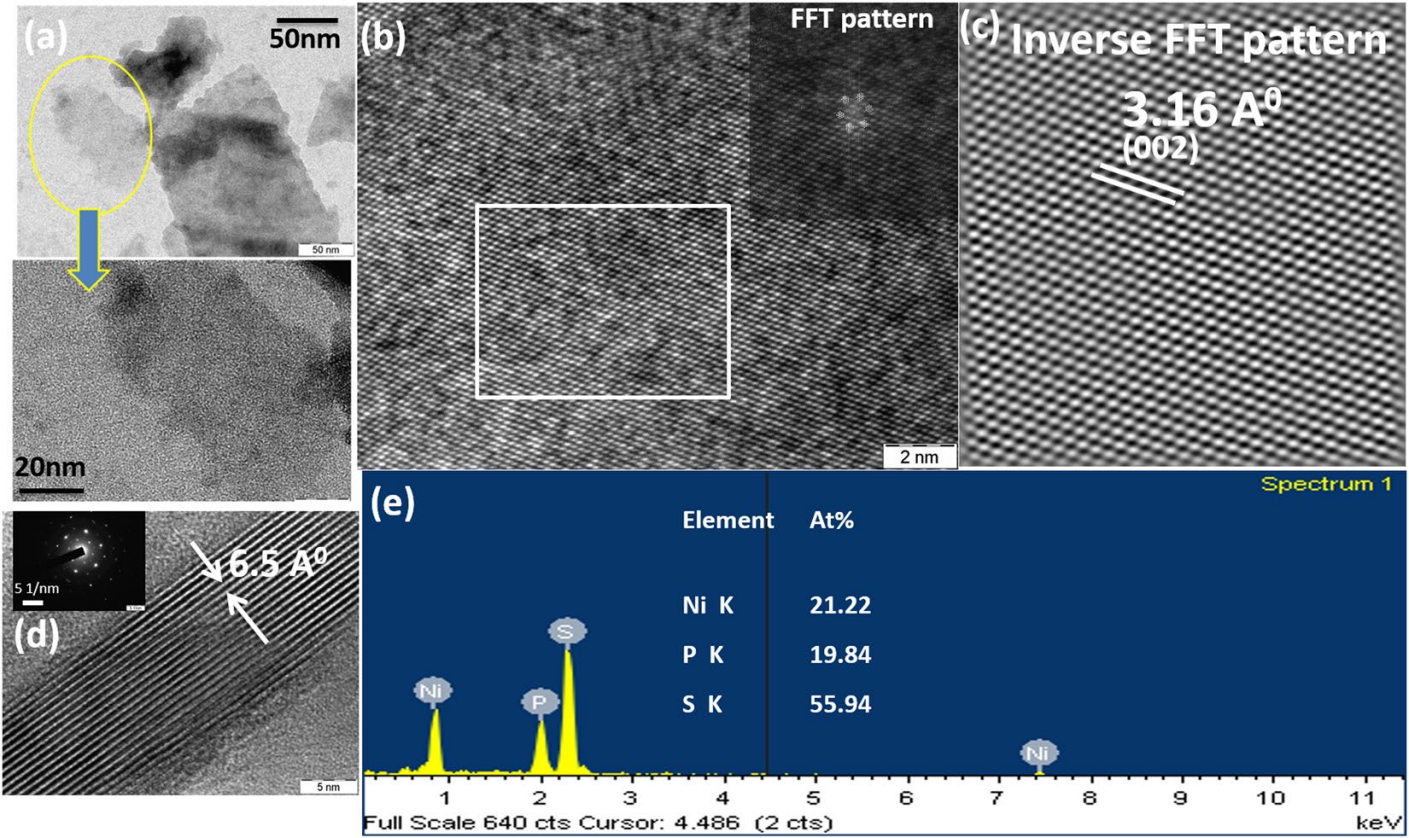
**(a**,**b**,**d**) High resolution TEM images of mechanically exfoliated few-layer NiPS_3_showing an interlayer spacing of 6.5 Å and the corresponding **(c)** FFT and **(e)** EDS mapping show the elemental ratio of Ni, P and S to be ~1:1:3.

The trans conductance (g_m_) value of NiPS_3_ obtained from the fabricated FET devices is 2.5 µS/cm. The TMDs, on the other hand show values of 0.5–3 µS/µm for back-gated devices^16^. The morphology and microstructure of exfoliated NiPS_3_ nanosheets are given in Fig. 2. Spatially resolved EDS elemental mapping of Ni, P and S elements reveals a ratio of 1:1:3 as expected with uniform distribution of the elements obtained over the entire surface. The HRTEM image shows well-resolved lattice fringes along (002) plane, confirming the quality of the crystalline nanosheet (Fig. 2b). The selected area electron diffraction (SAED) pattern displays a single set of diffraction spots, further confirming the oriented nature of the exfoliated nanosheets (Fig. 2c).

The low cleavage energy^41^ reported for NiPS_3_ makes it possible to fabricate devices containing few to several layers of the material. The bulk crystals are exfoliated by mechanical means (Fig. S2) as reported for graphene-based materials^54^. The devices used in the present study are prepared by transferring the material onto pre-fabricated contact pads on highly doped silicon as the back gate with 230 nm SiO_2_ dielectric by standard mechanical exfoliation method using scotch tape. The source-drain metal contacts are given using 10/60 nm ITO/Au. The flakes in the devices have been characterized using optical microscopy, Raman spectroscopy (with excitation wave length of 514 nm) and the thickness is measured using atomic force microscopy (AFM). Several micron-sized, large flakeswith thicknesses ranging from 1.5–10 nm have been achieved in the devices. The characteristics discussed the present study are for devices with varying thicknesses of 1.5–60 nm consisting of 2–75 layers.

**Figure 3:**
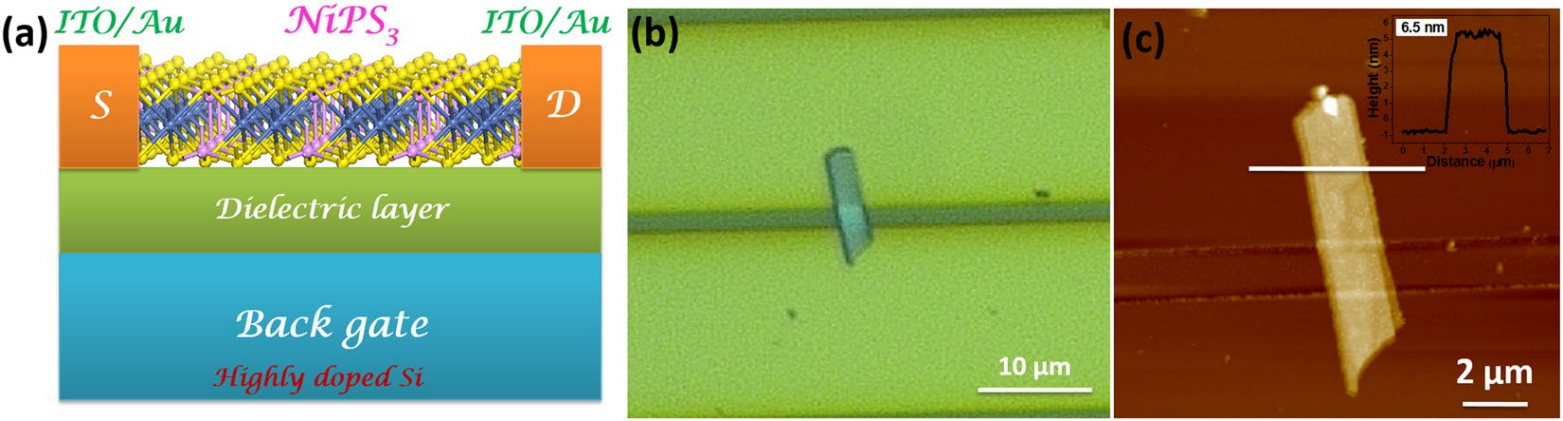
(**a)** Schematic of few-layer NiPS_3_ field effect transistor. (**b)** Optical micrograph of a typical NiPS_3_ device. (**c)** Atomic force microscopic image of 6.5 nm NiPS_3_ flake on Si/SiO_2_ with ITO/Au contact pads and the corresponding height profile measured across NiPS_3_ flake.

The back gated field effect transistors (FETs) have been fabricated and the characteristics followed. The data given below (Fig. 3) is for a channel length of 2.5 µm. The schematic of the FET along with the optical image, AFM picture and the height profile are shown in Fig. 3. It is clear that a large size single flake is present within the channel and the height is measured to be ~6.5 nm. This corresponds to 6 to 8 layers based on the single layer thickness of around 8 to 11 Å^40^. Standard transistor measurements have been carried out in vacuum (~10^−5^ mbar) and to ensure reproducibility, we have carried out measurements using several tens to hundreds of different devices.

**Figure 4:**
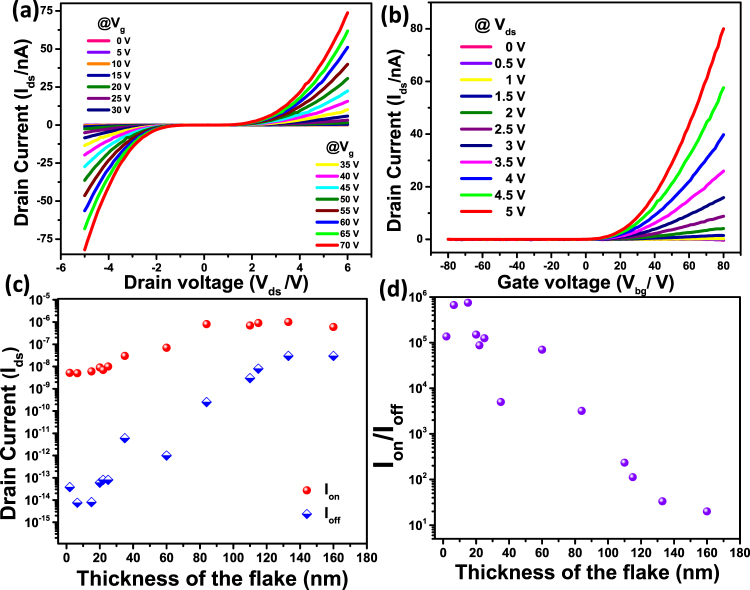
Electronic properties of few-layer NiPS_3_. (**a)** Drain current versus drain voltage for various Vg values as given in the figure, at 25 °C. (**b)** Drain current verses back gate voltage for V_ds_ ranging from 0 to 5 V. The FET characteristics obtained for a flake thickness of 6.5 nm NiPS_3_. (**c)** Thickness dependent I_on_ (red) and I_off_ (blue) currents. (**d)** Thickness dependence of I_on_/I_off_ ratio.

**Figure 5:**
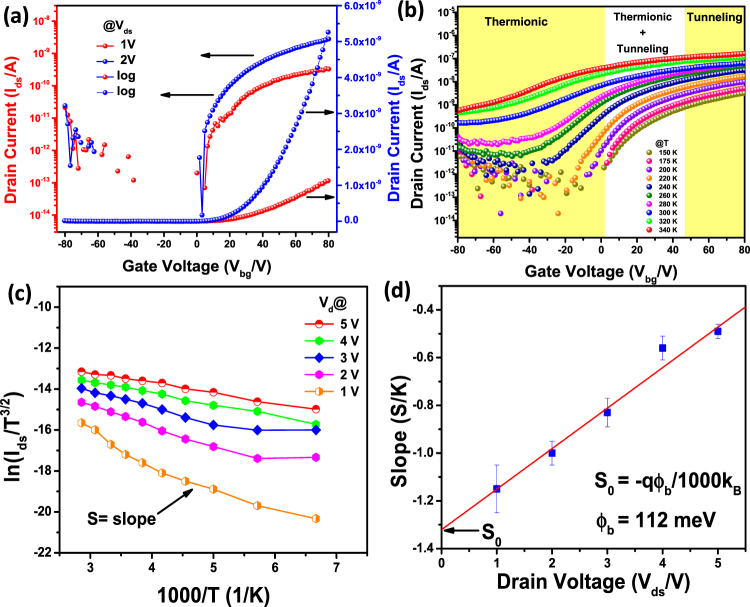
(**a)** Source-drain current as a function of gate voltage. (**b)** Temperature dependent transfer characteristics of the device. (**c)** Arrehenius plots of ln(I_ds_/T^3/2^) vs 1000/T. (**d)** Slope extracted from Fig. 5c as a function of V_ds_. Ф_B_ is derived from the y-intercept, S_0_.

The on-off ratio, threshold voltage, carrier type and mobility have been evaluated. The output characteristics of the devices, source-drain voltage (V_ds_) vs. source-drain current (I_ds_) at different gate voltages are shown in Fig. 4. The non-linear behavior possibly arises due to the schottky barrier contact with Au metal. The current increases with increasing positive gate voltage, suggesting n-type semiconducting behavior. The on-off ratio estimated based onthe transfer characteristics is ~10^3^–10^5^ for most of the devices (Fig. 5) at 25 °C. The field effect mobility is extracted from the I_ds_-V_bg_ curve using the following expression^5^.

1 $$\mu =[\frac{{d}{{I}}_{{ds}}}{{d}{{V}}_{{ds}}}]\times [\frac{{L}}{{W}{{C}}_{{i}}{{V}}_{{ds}}}]$$

where μ is the mobility, W (3 μm) is the channel width, L (2.5 μm) is the channel length and C_i_ (1.5 × 10^−4^ F/m^2^) is the capacitance between the channel and the back gate per unit area (C_i_ = ε_o_ε_r_/d; ε_o_ = 8.85 × 10^−12^ F⋅m^−1^; ε_r_ = 3.9; d = 230 nm). The mobility values are determined to be ~0.5–1 cm^2^/Vs. It is low as compared to several devices known in the literature. However, it is possible that poor contact with the metal electrode may be responsible as reported for MoS_2_ and WSe_2_^55,56^. The mobility may be improved further by using high-K dielectric materials in top gated devices and is being presently studied.

A threshold voltage of 10 V is estimated by extrapolating the transfer characteristics. The I_on_/I_off_ ratio as a function of thickness of the material is shown in Fig. 4d and it is observed that for thicknesses less than 60 nm, the I_on_/I_off_ ~ 10^5^ and it is of the order of 10^4^–10^2^ when the thickness is in the range 60–100 nm. Further increase in thickness leads to small I_on_/I_off_ ratio. This indirectly points to depletion length of ~100 nm for the present device. The I_on_/I_off_ ratio for MoS_2_ has been reported to be in the range of 10^7^–10^1^ for thicknesses varying from few nm to 250 nm^57^. The relationship between the depletion length with various parameters of the material is given below (Equation 2),

2 $${{\rm{W}}}_{{\rm{m}}{\rm{a}}{\rm{x}}}{\rm{=}}\sqrt{\frac{{\rm{4}}{\rm{k}}{\rm{T}}{{\rm{\varepsilon }}}_{{\rm{s}}}{{\rm{\varepsilon }}}_{{\rm{r}}}}{{{\rm{q}}}^{{\rm{2}}}{{\rm{N}}}_{{\rm{d}}}}{\rm{l}}{\rm{n}}\frac{{{\rm{N}}}_{{\rm{d}}}}{{{\rm{n}}}_{{\rm{i}}}}}$$

where, k is the Boltzmann constant, T is absolute temperature, q is elementary charge, ε_r_ is the vacuum permittivity, ε_s_ is the relative dielectric constant of NiPS_3_ (~9.2)^58^, n_i_ is intrinsic carrier concentration 6 × 10^5^ cm^−3^, and N_d_ is unintentional doping level. NiPS_3_ is known to be an intrinsic semiconductor with low conductivity^59^. For a depletion length of ~100 nm, the N_d_ works out to be 1.5 × 10^17^ cm^−3^ that points to certain level of doping in the material. It should be noted that NiPS_3_ possesses [P_2_S_6_]^4−^ clusters that leads to ‘ionic’ interactions with Ni^2+^ in the material^60^. The contributions of thermionic emission current component (I_thermionic_) and the thermally assisted tunneling current component (I_tunneling_) under different gate voltages indicate band bending at the metal - semiconductor interface. The Schottky barrier height (SBH) at the interface has been determined based on thermionic emission model using temperature dependent transport behavior. Figure 5b shows the devicecharacteristics at different temperatures and the change in current with the gate voltages is plotted (Fig. 5c) to extract the SBH using the following relationship (Equation 3).

3 $${{\rm{I}}}_{{\rm{d}}{\rm{s}}}{\rm{=}}{\rm{A}}{{\rm{A}}}^{{\rm{\ast }}}{{\rm{T}}}^{{\rm{3}}{\rm{/}}{\rm{2}}}{{\rm{e}}}^{[{\rm{-}}\frac{{\rm{q}}}{{\rm{k}}{\rm{B}}{\rm{T}}}({\rm{\Phi }}{\rm{B}}{\rm{-}}\frac{{{\rm{V}}}_{{\rm{d}}{\rm{s}}}}{{\rm{n}}})]}$$

where A is contact area of the junction, A* is the Richardson constant, q is magnitude of electron charge, Ф_B_ is the Schottky barrier height, k_B_ is Boltzmann constant, n is ideality factor, V_ds_ is drain-source voltage and T is the temperature. For the device shown in Fig. 3, the values of ln(I_ds_/T^3/2^) are plotted against 1000/T at different V_ds_ as shown in Fig. 5c. The slope at each bias is determined and plotted as a function of source-drain bias as shown in Fig. 5d. The intercept observed in Fig. 5b is determined (S_0_). Using the equation 4,

4 $${{\rm{S}}}_{0}=-\,\frac{{\rm{q}}{\rm{\Phi }}{\rm{B}}}{1000{{\rm{K}}}_{{\rm{B}}}}$$

the Schottky barrier height is calculated to be 112 meV for the 8 layer device of NiPS_3_. This value is similar to that observed for transition metal chalcogenide based devices^21,61^. Based on the small SBH determined from the transfer behavior, it may be expected that FET would show n-type unipolar behaviour and consequently the barrier height for holes is high [(band gap − SBH) for electrons].

### FET devices based on bi-layer and bulk NiPS_3_

The output characteristics of the bi-layer NiPS_3_ device, source-drain voltage (V_ds_) vs. source-drain current (I_ds_) at different gate voltages are shown in Fig. S3. The current amplitude increases with increasing positive gate voltage as observed for the n-type behavior of eight-layer device. However, the current values for the bi-layer NiPS_3_ device are quite low as compared to the multilayer device. It has been reported by Kim and co-workers^62^ that density of states for multilayer MoS_2_ is several times larger than that of single layer MoS_2_ thus making multilayers attractive for device applications with considerable drain currents. The on-off ratio estimated based on the transfer characteristics is ~10^5^ for the bi-layer devices and the mobility has been estimated to be ~0.5 cm^2^/Vs. The variation of mobility as a function of thickness of the flake (Fig. S4) is similar to the observations on reported TMD-based devices^63^. This is possibly due to scattering caused by extrinsic charge impurity that decreases as the number of layers increases, as reported for MoS_2_^63^. The transport behavior for the device with 60 nm thick flake (Fig. S5) is similar to that observed for other thicknesses. The current values are large with on-off ratio and mobility of ~10^2^–10^3^ and ~3.5 cm^2^/Vs respectively.

### DFT studies

**Figure 6:**
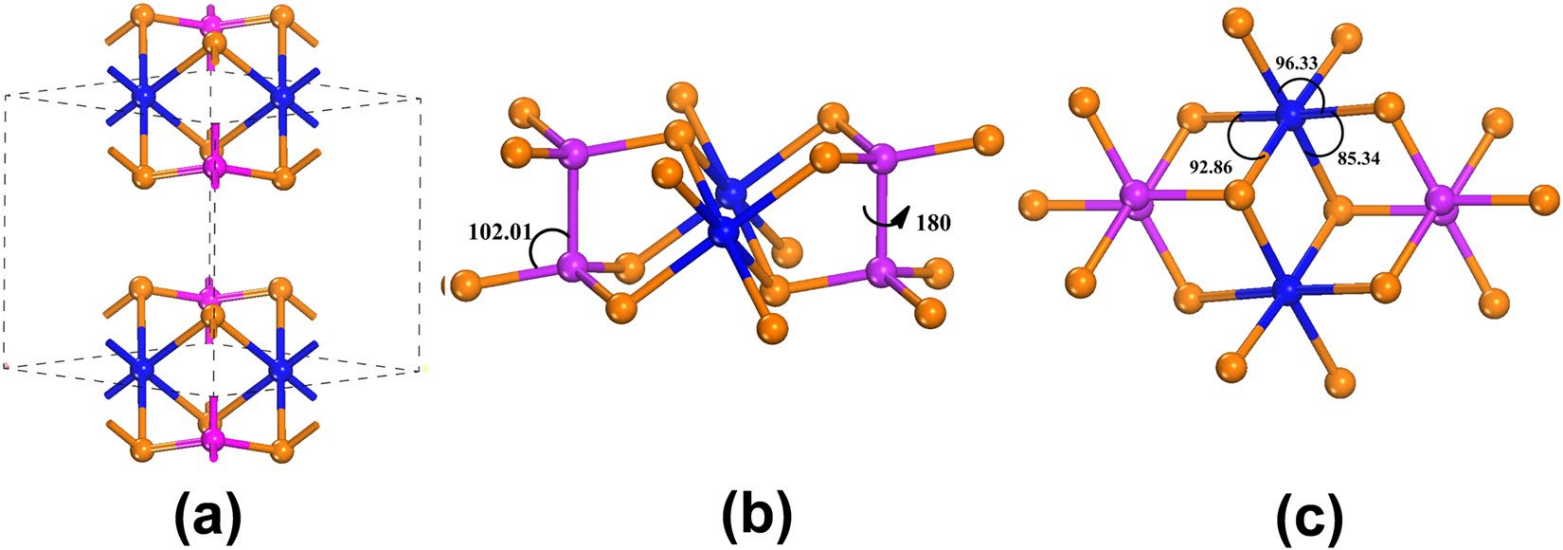
(**a)** NiPS_3_ crystal structure showing various atom positions and van der Waals gap. (**b)** P_2_S_6_^4−^ units showing the dihedral angle of S-P-P-S and the angle of S-P-P. (**c)** NiS_6_^10−^ unit with different bond angles. Blue, pink and brown colours represent nickel, phosphorus and sulphur atoms respectively.

Density functional theory (DFT) calculations have been performed to decipher the electrical transport behavior of NiPS_3_. The crystal structure of NiPS_3_ (Fig. 6a) illustrates that the (P_2_S_6_)^4−^ units are in staggered configuration where all sulphur atoms are coordinated to Ni in distorted octahedral environment and the van der Waals interlayer distance between S…S is 4.422 Å. The optimized geometry illustrates that the (P_2_S_6_)^4−^ units show dihedral (angle involving S-P-P-S structure) angle of 180°. The six S-P-P angles in the (P_2_S_6_)^4−^ unit being almost equal (Table S1), reveals that there is no strain present in the P_2_S_6_ clusters even when Ni is present in the lattice (Fig. 6b). On the contrary, the NiS_6_^10−^ units clearly reveal distortions in the structure. The distortion parameters (distortion angle, denoted as σ^2^oct)^64–66^ are measured as proposed by Robinson *et al*.^67^. Based on the structure (Fig. 6c), it is seen that there are twelve different bond angles that can be measured and the values are given in the Supporting Information (Table S1). The average σ^2^oct determined for neutral NiPS_3_ is 26.02°.

The DOS calculations (Fig. S6a) confirm the presence of up and down spins in the frontier region. The atom projected DOS given in Figure S7 indicates that the 3d bands of Ni (spin-up) are well-mixed with the 3p bands of S confirming strong hybridization as shown in SI (Fig. S6b,d). The upper valence bands are comprised of 3d orbital of Ni and 3p orbital of S. Sulphur dominates the valance band region whereas nickel contribution is maximum at the conduction band region. The orbital projected DOS of individual atoms reveals the presence of large population of 3p orbital of S at the Fermi whereas the contribution by phosphorus is very minimum. This may be due to the strong P-P covalent bond that appears in the lower valance band (−5 to −7 eV) energy region (Fig. S6c).

The FET characteristics of transistors and the type of conduction (p- or n-type) will depend on the relative position of the frontier region with respect to the metal contacts. To understand this aspect, the structural modulation of NiPS_3_ by electron/hole doping has been studied. The parameters obtained for the optimized geometries of neutral and hole/electron doped (0.1e^−^ per atom) NiPS_3_ are given in the supporting information (Table S2 and S3 in SI). Same level of theory has been used to perform calculations in all the cases. Doping an electron (0.1e^−^ per atom) to NiPS_3_ results in increased (8.01 eV) van der Waals gap and while for hole doping, the gap is found to be reduced (3.20 eV).

**Figure 7:**
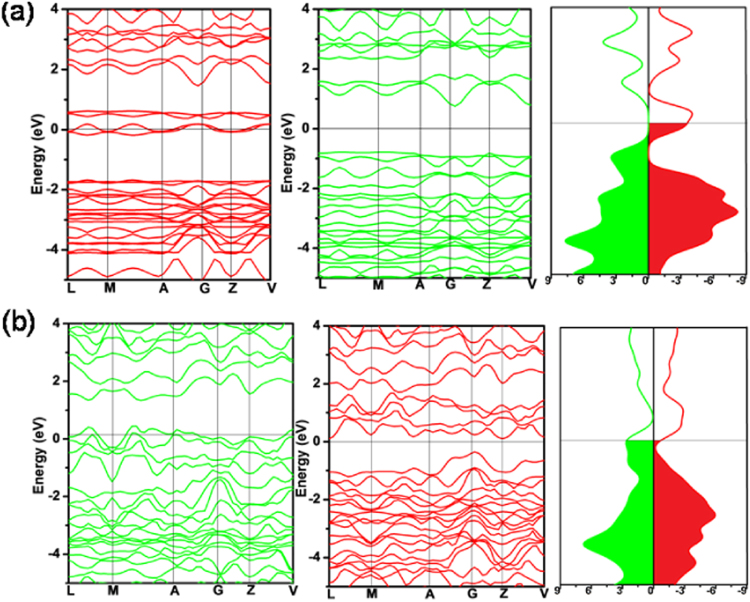
Band structure and total DOS of spin-up (green) and spin-down (red) configurations for (**a**) NiPS_3_ doped with 0.1 electron per atom, (**b**) NiPS_3_ doped with 0.1 hole per atom. The Fermi energy is set to zero.

Addition of an electron elongates the Ni-S bond length and the opposite effect is observed by hole doping. Very little changes are observed in P-S and P-P bond lengths during electron and hole doping. Further, it is seen that the addition of electron decreases the bond angle distortion in the NiS_6_ octahedra quite considerably to nearly zero (1.5°) while the hole doping increases the distortion angle by 12° from the neutral value of 26.02°. This suggests that electron doping leads to stable octahedral geometry around Ni. The changes observed in NiS_6_ units confirm the earlier prediction based on DOS calculations that the doping affects the environment around Ni and S. In the case of electron doping, spin up bands illustrate that the conduction band minimum is observed at G k point and the valance band maximum is found in between L and M k point with a band gap of 1.53 eV while the spin down structure becomes metallic (Fig. 7a). Similarly, the hole doping makes it metallic in nature for spin-up configuration and semiconducting for spin-down configuration (Fig. 7b) with an indirect band gap of 0.47 eV. The results show that NiPS_3_ changes from semiconducting to metallic nature tuned by electron/hole doping. Magnitude of band gap may also indirectly indicate the stability of materials upon doping. This can be related to the spin flip-gap related stability on doping (Fig. S1).

The atom projected DOS for individual atoms with and without electron/hole doping are shown in Fig. S7. Ni dominates at the Fermi for electron doping while S dominates for hole doping. Interestingly, for electron doped structure, significant increase in the population of Ni is observed near Fermi as compared to neutral NiPS_3_. It has moved to lower energy region indicating that the structure stabilizes with electron doping. The Ni population decreases near Fermi and minor reduction at the valance band region is observed for the hole doped material. In the case of S, major changes occur only during hole doping. The above results indicate that the electron doping affects Ni and hole doping affects sulphur and consequently the NiS_6_ unit. This is supported by the lowered bond angle distortions during electron doping thus leading to stable NiS_6_^10−^ octahedral environment.

### Summary

The present study has shown the possibilities of fabricating field effect transistors using layered phosphochalcogenides, NiPS_3_. The FET characteristics show n-type behavior with on/off ratio of 10^3^–10^5^. The DFT studies have predicted the transport characteristics and are experimentally verified. The phosphochalcogenides with magnetically active centers such as Ni, Co and Mn open ways to flip the spin behavior under magnetic field.

## Experimental Section

### Synthesis of NiPS_3_ crystals

Single crystals of NiPS_3_ have been synthesized by chemical vapor transport (CVT) technique using iodine as the transporting agent. Pure elements (99.99%) of nickel, phosphorous and sulphur (Aldrich) in stoichiometric proportions with I_2_ (2 mg/cc) were sealed in an evacuated quartz ampoule. After several attempts, the optimum reaction conditions were arrived at, with hot zone temperature of 950 °C and cold zone of 850 °C that result in high quality, large sized crystals of NiPS_3_. The schematics of CVT growth setup and the parameters used are given in the supporting information (Fig. S8, S9).

### Characterization

The physicochemical nature of the crystals were identified using X-ray diffraction (XRD) (Philips, PAN analytical, with Cu-Kα radiation), transmission electron microscopy (TEM, JEOL 2100 F operating at 200 kV), Raman spectroscopy (LabRAM, Horiba, France, with excitation wavelength of 514.5 nm and 50x long working distance objective) and atomic force microscopy (AFM, Veeco, NanoscopeIVa Multimode AFM, with silicon nitride, Si_3_N_4_ probes of length 130 µm, width 35 µm, resonance frequency, 270 kHz and force constant, 4.5 N/m). The samples for TEM measurements were prepared by dispersing few-layer NiPS_3_ colloids onto carbon-coated copper grid and dried under vacuum. The electrical measurements were performed under high vacuum (<5 × 10^−5^) using Agilent B1500 semiconductor parametric analyzer. Devices were prepared by transferring the material onto pre-fabricated contact pads (Fraunhofer IPMS, Germany) on highly doped silicon with 230 nm SiO_2_ dielectric using standard mechanical exfoliation method. Source-drain metal contacts were given using 10/60 nm ITO/Au. Highly doped silicon act as the back gate. It was observed that the lithography procedure wherein the sample was exfoliated on Si/SiO_2_ surface and subsequently depositing the contact pads on the pre-marked areas led to sample deterioration due to the use of solvents during the process.

### Computational methodology

Geometrical optimization calculations for all extended structures has been performed using VASP code^68,69^ with plane wave basis truncated at a kinetic energy of 500 eV. The projector augmented wave (PAW) scheme as incorporated in the Vienna ab initio simulation package (VASP) is used in the study (Supporting Information). Density of states and band structure calculations have been performed followed by optimization using CASTEP package^70^ including LDA + U approximation. Calculations have been performed for neutral NiPS_3_ and electron/hole doping separately, and the values are tabulated in Table S1 (Supporting Information).

## Electronic supplementary material

Supplementary Information
