# Supplementary material for: Field Effect Transistor Based on Layered NiPS3
Source: Sci Rep. 2018 Jun 5;8:8586. doi: 10.1038/s41598-018-26522-1 (PMC5988702; doi:10.1038/s41598-018-26522-1)
Supplement: Supplementary file 1 — Supplementary Information [file 41598_2018_26522_MOESM1_ESM.doc]

Supplementary Information

Field Effect Transistor Based on Layered NiPS_3_

Ramesh Naidu Jenjeti, Rajat Kumar, Muthu Austeria P* and S Sampath*

Department of Inorganic and Physical Chemistry

Indian Institute of Science

Bangalore 560012, India

**Figure S1a.** Schematic diagram for different spin polarized BMS material upon electron/hole doping. Various symbols are explained in the text. Adapted from Li, X et.al.^1^

**Figure S1b.** Schematics of the FET device showing the values of Δ1, Δ2 and Δ3 along with the on current/off current ratio versus the thickness of the flake. Various symbols are explained in the text.

**Mechanically exfoliated NiPS_3_ flakes**

**Figure S2.**(a, d, g) Optical images of mechanically exfoliated NiPS_3_ of different layer thicknesses. (b, e) and h correspond to SEM images. The AFM images of different layers along with height profiles. Scale bar is 10 μm.

**FET devices of bi – layer and bulk NiPS_3_**

**Figure S3**(a). Optical microscopic image of bi-layer NiPS_3_ device. Inset shows AFM image along with height profile. Field effect transistor data of bi-layer NiPS_3_ (b) I_ds_-V_ds_ and (c) I_ds_-V_bg_.

**Thickness dependent mobility of NiPS_3_**

**Figure S4.** Mobility extracted from FET devices for various thickness of the flakes.

**Figure S5**(a). Optical microscopic image of 60 nm NiPS_3_ device. (b) AFM image along with the height profile (c) FET data, I_ds_-V_ds_ and (d) I_ds_-V_bg_ behviour.

**Methodology:**

Geometrical optimization calculations for all extended structures has been performed using VASP code^(64, main text)^ with plane wave basis truncated at a kinetic energy of 500 eV. The projector augmented wave (PAW) scheme as incorporated in the Vienna ab initio simulation package (VASP) isused in the study. The Monkhorst and Pack scheme of k point sampling is used for integration over the first Brillouin zone. A 3x3x3 grid for k-point sampling for geometry optimization of unit cells, and an energy cut-off of 500 eV are consistently used in our calculations. For bilayer calculations in neighbouring cells in the vertical direction are separated by a vacuum region of at least 20 Å. Good convergence is obtained with these parameters and the total energy was converged to 2X3 1025 eV/atom. The error bar or uncertainty of the DFT calculations is less than 5 meV. Geometry optimizations were performed with full optimization of the unit cell parameters and ionic positions within the lattice symmetry constraints to arrive at the well converged structures. Single point energy calculations done with optimized extended structure by using CASTEP code available within the Materials Studio suite^(65, main text)^ We employed the semiempirical LDA+U approach to treat the transition metal of Ni 3d electrons. A generalized gradient approximation (GGA) was chosen to deal with the exchange and correlation potential in the calculations. The GGA functional form Perdew–Burke–Ernzerhof (PBE)[^2^](#_ENREF_17) was used. Vanderbilt Ultrasoft pseudo potentials and Monkhorst–Pack k-point[^3^](#_ENREF_18) mesh with separation between k-points set (3x3x3) at 0.04Å is used for band structure and Density of States (DOS) calculations. All DFT calculations will underestimate band gap. So, we used scissor operator (1.0 eV) for band structure and density of states (DOS) calculations, to obtain the experimental band gap.

**Figure S6**(a). Individual orbital projected density of states for spin -up and spin -down configurations in bulk NiPS_3_ (b) orbital projected DOS for spin-up and spin down for Ni. (c) phosphorus d) sulphur atoms.

**Figure S7.** Atom projected DOS for a) sulphur b) nickel and c) phosphorus with and without electron/hole doping.


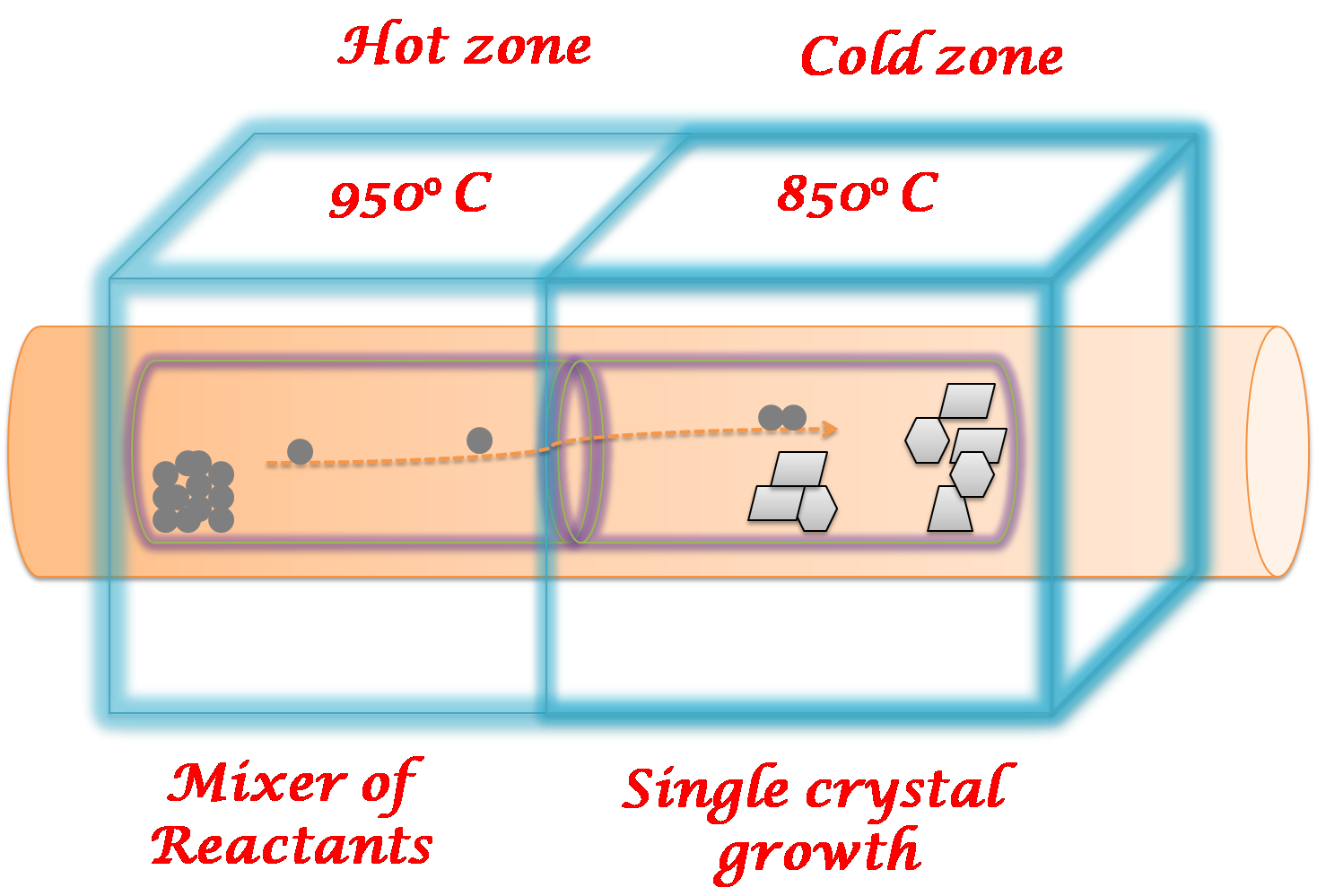


**Figure S8.** Schematic diagram of CVT growth method for single crystals of large sized NiPS_3_.

**Figure S9.** Photographic images of NiPS_3_ grown by CVT method.

**Gate leakage:**

After fabrication of the device, we performed the experiment for checking the gate leakage and found that most of the devices show negligible gate leakage currents of less than 10 pA/μm in ±80V voltage range. Leakage currents through the bottom gate for the sweep shown in figure below.

**Table S1. Various S-Ni-S bond angles observed for neutral bulk NiPS_3_, electron doped and hole doped NiPS_3_ (see text for details).**

| Bond angle no. | S-Ni-S bong angle, (deg.) | | |
| --- | --- | --- | --- |
|  | Bulk NiPS_3_ | Hole doped NiPS_3_ | Electron doped NiPS_3_ |
| 1 | 96.553 | 90.633 | 91.551 |
| 2 | 85.339 | 84.699 | 88.888 |
| 3 | 92.862 | 100.414 | 88.871 |
| 4 | 85.318 | 84.699 | 90.696 |
| 5 | 96.653 | 99.223 | 91.843 |
| 6 | 85.262 | 85.080 | 88.907 |
| 7 | 92.893 | 85.635 | 90.347 |
| 8 | 85.262 | 90.475 | 88.907 |
| 9 | 96.553 | 99.223 | 91.551 |
| 10 | 92.862 | 90.475 | 90.696 |
| 11 | 85.339 | 85.635 | 88.871 |
| 12 | 85.318 | 85.080 | 88.888 |

**Table S2. Geometric parameters obtained using DFT for layered neutral NiPS_3_ material. Experimentally observed**[**^4^**](#_ENREF_15) **crystal structure parameters are shown for comparison.**

| Material | Bond length (Å) | | | Bond angles (°) | | | | Lattice Parameters |
| --- | --- | --- | --- | --- | --- | --- | --- | --- |
|  | P-S | Ni-S | P-P | Ni-S-Ni | Ni-S-P | S-P-S | S-P-P |  |
| NiPS_3_ neutral  (Exp.) | 2.0135  2.0165 | 2.4893 | 2.1536 | 84.93 | 102.73 | 114.30  114.55 | 104.03  103.78 | a,b,c = 5.8199, 10.0825, 6.627  α,β,γ = 90, 107.10, 90 |
| NiPS_3_ neutral  (Theory) | 2.0579  2.0593 | 2.4432 | 2.1792 | 87.14 | 103.33 | 115.69  115.72 | 102.01  102.20 | a,b,c=  5.8362, 10.1052, 10.4699  α,β,γ =90, 138.07, 90 |

**Table S3.** Geometric parameters obtained using DFT for NiPS_3_ with hole and electron doping.

| Material | Bond length ( Å) | | | Bond angle (°) | | | | Lattice parameters |
| --- | --- | --- | --- | --- | --- | --- | --- | --- |
|  | P-S | Ni-S | P-P | Ni-S-Ni | Ni-S-P | S-P-S | S-P-P |  |
| NiPS_3_ 0.1electron doping per atom | 2.0424  2.0574 | 2.2764 | 2.1554 | 89.37 | 102.65 | 117.26  117.01 | 98.83 | 5.5927, 9.6635, 9.2097  α,β,γ =90, 144.17,90 |
| NiPS_3_ 0.1hole doping per atom | 2.0720  2.0704 | 2.4943 | 2.2119 | 89.30 | 106.95 | 117.07  117.10 | 99.91 | 6.0672,  10.4908,  12.7421  α,β,γ =90,  129.47, 90 |

**References**

1. Li, X., Wu, X., Li, Z., Yang, J. & Hou, J. G. *Nanoscale* **4**, 5680-5685 (2012).
2. Perdew, J. P., Burke, K. & M. Ernzerhof, *Phy. Rev. Lett.* **77***,* 3865-3868 (1986).
3. Monkhorst, H. J. & Pack, J. D. *Phy. Rev. B* **13,** 5188-5192 (1976).
4. Fragnaud, P. B. R., Prouzet, E. & Deniard, P. *Mat. Res. Bull.,* **28**, 337-346 (1993).
